# Degeneracy and Photon Trapping in a Dissipationless Two-Mode Optomechanical Model

**DOI:** 10.3390/e26010087

**Published:** 2024-01-19

**Authors:** Thiago Alonso Merici, Thiago Gomes De Mattos, José Geraldo Peixoto De Faria

**Affiliations:** 1 Programa de Pós-Graduação em Modelagem Matemática e Computacional, Centro Federal de Educação Tecnológica de Minas Gerais (CEFET-MG), Av. Amazonas 7675, Belo Horizonte 30510-000, MG, Brazil; thiago.merici@ifmg.edu.br (T.A.M.); tgmattos@cefetmg.br (T.G.D.M.); 2 Departamento de Física, Centro Federal de Educação Tecnológica de Minas Gerais (CEFET-MG), Av. Amazonas 7675, Belo Horizonte 30510-000, MG, Brazil; 3 Departamento de Matemática, Centro Federal de Educação Tecnológica de Minas Gerais (CEFET-MG), Av. Amazonas 7675, Belo Horizonte 30510-000, MG, Brazil

**Keywords:** finite systems, optomechanical systems, Dicke model

## Abstract

In this work, we theoretically study a finite and undamped two-mode optomechanical model consisting of a high quality optical cavity containing a thin, elastic, and dielectric membrane. The main objective is to investigate the precursors of quantum phase transition in such a model by studying the behavior of some observables in the ground state. By controlling the coupling between membrane and modes, we find that the two lowest energy eigenstates become degenerate, as is indicated by the behavior of the mean value of some operators and by other quantifiers as a function of the coupling. Such degenerate states are characterized by a coherent superposition of eigenstates describing one of the two modes preferentially populated and the membrane dislocated from its equilibrium position due the radiation pressure (Schrödinger’s cat states). The delocalization of the compound system photons+membrane results in an increase in fluctuations as measured by Robertson-Schrödinger uncertainty relations.

## 1. Introduction

Optomechanical systems are characterized by the coupling between radiation and matter. In general, this coupling is established by the interaction of radiation pressure with some mechanical degree of freedom [1,2,3,4]. The increasing development of techniques in experimental quantum optics has made it possible to control and handle the interaction between electromagnetic fields and macroscopic mechanical devices at the quantum scale [2].

An example of a simple optomechanical system consists in a moving mirror which is coupled to a spring and is hit by photons. In order to optimize the interaction between the light and the mechanical oscillator, photons can be trapped in an optical cavity in which one of the mirrors is free to oscillate. Photons undergo multiple reflections between the mirrors, increasing the interaction time with the moving mirror, thus increasing the radiation pressure on it. In an optical cavity with a moving mirror, the natural frequency of the electromagnetic mode is determined by the size of the cavity. This feature is known as parametric coupling [2,5].

Optomechanical systems have attracted increasing attention in recent years. Many experiments and theoretical models have been proposed in order to better understand fundamental aspects of the interaction between radiation and matter, more specifically those associated with macroscopic mechanical systems and electromagnetic fields at the quantum scale. As examples, we cite quantum non-demolition measurements of a mechanical quadrature [6,7], photon antibunching [8], cooling of mechanical modes to their fundamental state [9,10,11,12], optomechanically induced transparency [13], macroscopic quantum superposition in mechanical systems [14,15,16], quantum-coherent coupling of a mechanical oscillator to an optical cavity mode [17], quantum entanglement between electromagnetic fields and a mechanical oscillator [18], and quantum sensors of mechanical motion [19,20,21]. Moreover, optomechanical systems have great potential for practical applications, such as radio-frequency oscillators [22,23], optical memories [13], high sensitivity probes of acceleration [24], and magnetic fields [25].

**Figure 1:**
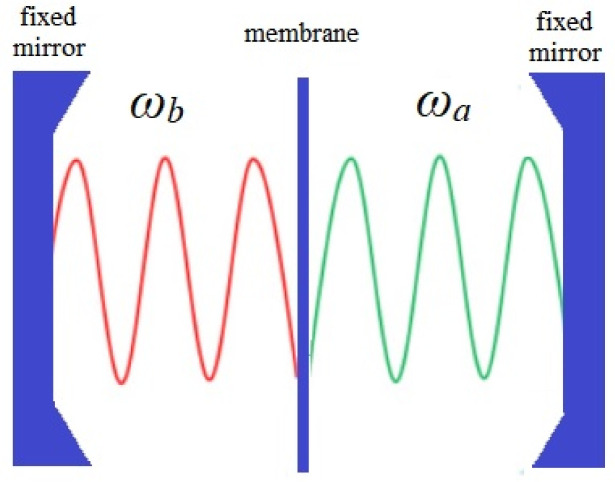
Setup of the two-mode optomechanical system. A thin, elastic, and dielectric membrane is placed in the middle of a high-*Q* optical cavity. As a result, two modes are formed: one at left and the other at right position of the membrane, with frequencies $\omega_{a}$ and $\omega_{b}$. If the equilibrium position of the membrane is symmetric with respect to the mirrors, we can consider $\omega_{a}=\omega_{b}=\omega_{0}$. This system allows photons to be transmitted from one mode to another at a rate *g*. The system is completely isolated and the membrane is frictionless: losses or pumping of photons and phonons to or from the environment are not considered.

In this work, we theoretically study a model for a high quality optical cavity with fixed mirrors and an elastic dielectric membrane within it, as depicted in Figure 1. Two electromagnetic modes are produced inside the cavity, one on each side of the membrane. As the membrane’s transmission amplitude is not null, the modes can exchange photons. Additionally, the radiation pressure causes the membrane to vibrate, affecting the frequencies of the modes on each side [26,27], as a consequence of parametric coupling.

This kind of two-mode optomechanical model can be treated as a realization of the *N*-atoms Dicke model by properly mapping the field operators into pseudospin operators. Accordingly, the two modes are mapped into the atomic component of the Dicke model whereas the membrane is associated with the corresponding field component [27]. In thermodynamic limit, such models are known to present quantum phase transition (QPT) and spontaneous $Z_{2}$-symmetry breaking [27,28,29,30] depending on the value of parameters. QPT and symmetry breaking are phenomena usually assigned to macroscopic systems and the mentioned thermodynamic limit starts from a finite system, i.e., a system formed by a finite number *N* of components occupying a finite volume *V*, and take the limits N→∞ and V→∞, so that N/V is kept finite. However, some attention has been paid to the precursors of these macroscopic phenomena in their corresponding finite systems [31,32,33,34,35,36]. Such precursors are related with changes undergone by the finite system as some parameter or potential is varied. This is the situation studied here, since we consider a two-mode optomechanical model with a finite number of photons.

In this work, we observe how the ground state of the corresponding Hamiltonian of the “membrane-in-the-middle” optomechanical model is affected as one of the system parameters is varied. In general, it is expected that the appearance of some kind of non-analyticity in ground state will occur at the critical point. Such a non-analyticity can manifest itself in the mean values of some chosen observables—e.g., the imbalance in the photon number stored in the two modes—and in the fidelity susceptibility. For this case, there are two parameters of interest that could be used to access the precursors of QPT or symmetry breaking: the reflectivity of the membrane and the coupling between the field and the membrane resulting from the radiation pressure. We chose to keep constant the membrane reflectivity, whereas the coupling field-membrane is varied. This study takes into account a very idealized situation: the optomechanical system is perfectly isolated from the environment, i.e., photon leakage or the entry of thermal photons throughout the mirrors or dissipative forces on the membrane are not considered. Moreover, optical fields inside the cavity are not pumped by external sources. We recognize that such simplifications are not verified in experiments involving optomechanical devices. Despite this, the optomechanical system is studied in a full quantum frame, without resorting to semiclassical approximations or similar methods.

This work is organized as follows: in Section 2, we present the two-mode optomechanical model and map it into the Dicke model; in Section 3, we present the investigation of this model close to the appearance of non-analyticities in the ground state; and the last section is reserved for the final remarks and conclusion.

## 2. The Model

In this paper, we follow the schematic and nomenclature adopted in Ref. [27]. Consider a high-*Q* optical cavity of length *L* which has a highly reflective dielectric membrane placed equidistant between its two mirrors, as shown in Figure 1. The membrane is elastic and able to vibrate like a drum skin. We consider only two cavity modes in this study: one assigned to the right side, with frequency $\omega_{a}$, and one assigned to the left side of the membrane, with frequency $\omega_{b}$. We assume that these frequencies are equal when the membrane is localized at the center of the high-*Q* cavity, i.e., $\omega_{a}=\omega_{b}=\omega_{0}$. As an additional approximation, we assume the system is perfectly isolated. Thus, we do not take into account losses due to friction or imperfections of the mirrors. Moreover, the cavity modes are not fed by any kind of external pump.

As is shown below, this two-mode optomechanical model can be mapped into the well-known Dicke model, which describes the interaction between a number of two-level atoms with an electromagnetic field. Note that dissipation has been theoretically considered in studies of the Dicke model [37,38,39,40], based on mean field or semiclassical approaches. Such works unveil novel and interesting properties absent in the undamped case, e.g., if collective damping of atoms are taken into account, bicritical transitions or coexistence of two or more phases are predicted [37,38]. Experimental simulations of dissipative Dicke models were performed using ultracold atoms confined to high-finesse cavities [41,42]. The present work adopts the approximation of closed system that is not prevalent in quantum optomechanical models. However, we consider the case of a finite size system and we employ a full quantum mechanical approach as an initial characterization the two-mode optomechanical model. The studies in the dissipative Dicke model domain cited just above point out the inclusion of dissipation and optical pump as a natural extension of our work, and suggest the two-mode optomechanical model as a promising platform to realize novel phenomena, as hysteresis and multiphase transitions.

The membrane is modeled as a mechanical harmonic oscillator with natural frequency ω and undergoes displacement due to a radiation pressure proportional to the photon number difference (imbalance) between the right and left modes. Further, the frequencies of these modes instantaneously depend on the position of the membrane. Assuming that the mode frequencies depend linearly on the membrane displacement, the full quantum description of this model is given by the Hamiltonian

(1) $$\hat{H}=\frac{{\hat{p}}^{2}}{2m}+\frac{m\omega^{2}{\hat{x}}^{2}}{2}+\hbar g\left({\hat{a}}^{†}\hat{b}+{\hat{b}}^{†}\hat{a}\right)+\frac{2}{L}\hbar \omega_{0}\hat{x}\left({\hat{n}}_{a}-{\hat{n}}_{b}\right),$$

where *m* stands for the mass of the mechanical oscillator, and $\hat{x}$ and $\hat{p}$ are its position and linear momentum operators, respectively. Photon annihilation (creation) operators of the right and left cavity modes are respectively represented by $\hat{a}$ (${\hat{a}}^{†}$) and $\hat{b}$ (${\hat{b}}^{†}$), and ${\hat{n}}_{a}={\hat{a}}^{†}\hat{a}$ and ${\hat{n}}_{b}={\hat{b}}^{†}\hat{b}$ are their corresponding photon number operators. The operators $\hat{a}$, ${\hat{a}}^{†}$, $\hat{b}$, ${\hat{b}}^{†}$ satisfy the usual bosonic commutation relations, $\left[\hat{a},{\hat{a}}^{†}\right]=\left[\hat{b},{\hat{b}}^{†}\right]=1$ and $\left[\hat{a},\hat{b}\right]=0$. From now on, for the sake of brevity, we describe the right and left cavity modes as *a* and *b* modes, respectively. Finally, the coupling constant *g* is related to the reflectivity of the membrane, assumed close to unity. *g* measures the rate that the modes swap photons and is given by

(2) $$g=\frac{c\sqrt{2\left(1-r\right)}}{L},$$

where *c* stands for the velocity of light in the vacuum, the full cavity length *L*, and the membrane’s intensity reflectivity *r*, which in turn depends on the refractive index and the thickness of the membrane (for more information, see [26,43]). Defining the dimensionless operators $\hat{X}=\hat{x}\sqrt{m\omega /\hbar }$ and $\hat{P}=\hat{p}/\sqrt{m\hbar \omega }$, the phonon annihilation and creation operators are respectively given by $\hat{c}=1/\sqrt{2}\left(\hat{X}+i\hat{P}\right)$ and ${\hat{c}}^{†}=1/\sqrt{2}\left(\hat{X}-i\hat{P}\right)$. Additionally, the phonon number operator is defined by ${\hat{n}}_{c}={\hat{c}}^{†}\hat{c}$. Providing these definitions and establishing ${\hat{H}}^{\prime }=\frac{\hat{H}}{\hbar \omega }$ and the dimensionless parameters $g^{\prime }=g/\omega$ and $\lambda =\frac{2}{L}\sqrt{\frac{\hbar \omega_{0}^{2}}{m\omega^{3}}}$, the Hamiltonian in Equation (1) can be rewritten as

(3) $${\hat{H}}^{\prime }={\hat{n}}_{c}+2g^{\prime }{\hat{S}}_{x}+\sqrt{2}\lambda \left(\hat{c}+{\hat{c}}^{†}\right){\hat{S}}_{z}.$$

In the equation above, to establish the correspondence with the Dicke model, we use the pseudospin operators, defined as

(4) $${\hat{S}}_{x}=\frac{1}{2}\left({\hat{a}}^{†}\hat{b}+{\hat{b}}^{†}\hat{a}\right),$$

(5) $${\hat{S}}_{y}=\frac{1}{2i}\left({\hat{a}}^{†}\hat{b}-{\hat{b}}^{†}\hat{a}\right),$$

(6) $${\hat{S}}_{z}=\frac{1}{2}\left({\hat{n}}_{a}-{\hat{n}}_{b}\right).$$

The operators above obey the usual commutation relations of the SU(2) algebra, i.e., $\left[{\hat{S}}_{i},{\hat{S}}_{j}\right]=i\varepsilon_{ijk}{\hat{S}}_{k},$ with i,j,k take values in $\left\{x,y,z\right\}$. Here, $\varepsilon_{ijk}$ stands for the Lévi-Civita symbol.

The Hamiltonian in Equation (3) has similarities with the corresponding Hamiltonian of the Dicke model [44,45,46,47,48], except by the ${\hat{S}}_{x}↔{\hat{S}}_{z}$ swapping, which can be viewed as a rotation of the spin operators around the *y*-axis. Here, the membrane plays the role of the field and the *a* and *b* modes play the role of atoms in Dicke’s original model.

## 3. Degeneracy and Photon Trapping

The results presented in this section were obtained by the diagonalization of the Hamiltonian ${\hat{H}}^{\prime }$. The observable ${\hat{n}}_{a}+{\hat{n}}_{b}$ commutes with the Hamiltonian ${\hat{H}}^{\prime }$ and the quantum number *S* is associated to the total number of photons stored in both modes. In fact, since the eigenvalues of the photon imbalance operator ${\hat{S}}_{z}$ defined in Equation (6) runs from −S (all photons found in *b*-mode) to *S* (all photons in *a*-mode), the number of photons stored in the cavity is equal to 2S. Thus, when establishing a given value to *S*, we define a particular subspace of the global space of states characterized to collect the eigenstates of ${\hat{n}}_{a}+{\hat{n}}_{b}$ with the same eigenvalue 2S. When we refer to the ground state of ${\hat{H}}^{\prime }$ for a fixed *S*, we refer to the lowest energy eigenstate of ${\hat{H}}^{\prime }$ restricted to a particular subspace.

On the other hand, the number of phonons, represented by ${\hat{n}}_{c}$, is not preserved in the dynamics governed by the Hamiltonian ${\hat{H}}^{\prime }$. This fact imposes some challenges to the numerical diagonalization of ${\hat{H}}^{\prime }$, since the exact expansion of any of its eigenstates using the eigenvectors of ${\hat{n}}_{c}$ produces an infinity number of unknown coefficients. For diagonalization to be feasible, truncation in this expansion is imposed, limiting the number of eigenvectors of ${\hat{n}}_{c}$ in an expansion of an eigenstate of ${\hat{H}}^{\prime }$. We define this truncation as $N_{\max}$, and thus the basis used in the numerical diagonalization is the set ${\left\{|m,n_{c}\rangle \right\}}_{m=-S\ldots S,n_{c}=0\ldots N_{\max}}$. However, as discussed by Chen and coworkers in Ref. [49], the energy of the ground state of ${\hat{H}}^{\prime }$ does not converge quickly as the number of terms included in the ground state expansion increases.

### 3.1. Photon Imbalance Close to Critical Transition

We study the mean value of this observable, represented by the operator ${\hat{S}}_{z}$, evaluated in the ground state $|\Psi_{0}\rangle$ of the Hamiltonian given by Equation (3), $\langle {\hat{S}}_{z}\rangle =\langle \Psi_{0}|{\hat{S}}_{z}|\Psi_{0}\rangle$. The parameter λ that controls the coupling among the membrane and the modes is varied and $g^{\prime }$ is kept constant and equal to 1/2. As pointed out by Mumford and coworkers [27], in typical optomechanical systems, $g^{\prime }\gg 1$, even the intensity reflectivity *r* is very close to the unity. To reach $g^{\prime }\approx 1$, the natural vibration frequency of the membrane should be in the order of tens of MHz. This fact can impose some obstacles to experimental realization of this model. Such a proceeding is repeated for several values of the quantum number *S*. The numerical diagonalization of the Hamiltonian ${\hat{H}}^{\prime }$ is feasible by defining a truncation of the number of excitations (phonons) of the mechanical part, $N_{\max}$. To renormalize the coupling among membrane and modes, we define $u=\lambda \sqrt{S}$.

**Figure 2:**
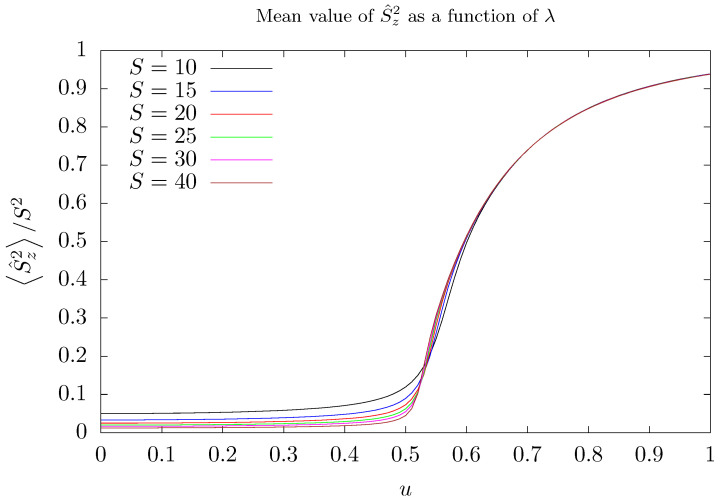
Mean value of the operator ${\hat{S}}_{z}^{2}$ (i.e., squared photon imbalance operator) as a function of *u* in the ground state of the Hamiltonian of Equation (3) for several values of the quantum number *S*: 10 (black), 15 (blue), 20 (red), 25 (green), 30 (magenta), and 40 (brown). For all curves, $g^{\prime }=1/2$ and $N_{\max}=5S$.

As the parameter λ is varied, the system undergoes a significant change: the two lowest energy eigenstates of ${\hat{H}}^{\prime }$ become degenerate. Degeneracy occurs for a critical value given by $\lambda_{1}=1/\left(2\sqrt{S}\right)$. This value can be determined using the corresponding classical Hamiltonian of ${\hat{H}}^{\prime }$ obtained taking the limit of the number of stored photons going to infinite. This evaluation can be found in Appendix A. The occurrence of such a degeneracy can be detected by the way the mean value of some observables changes as λ is varied. Figure 2 shows the mean value of the imbalance operator squared ${\hat{S}}_{z}^{2}$ as a function of *u*. As one can see, a change in $\langle {\hat{S}}_{z}^{2}\rangle /S^{2}$ is observed for $u_{1}\approx 1/2$, as *u* is increased: it goes from small positive values to a value close to 1, regardless of the number of photons stored in the cavity.

**Figure 3:**
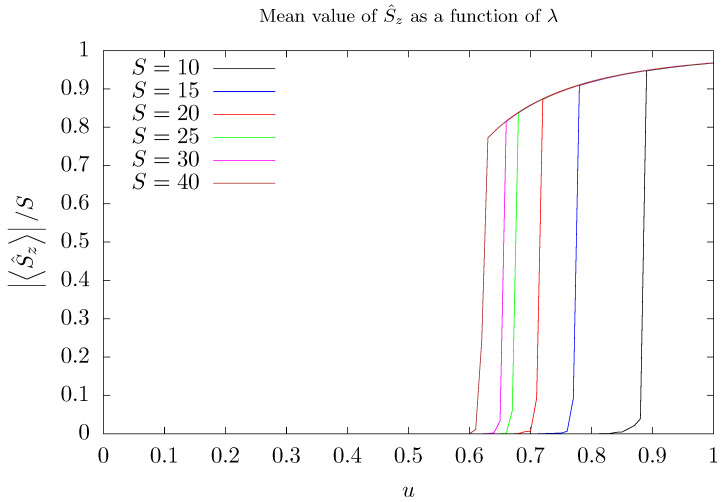
Modulus of mean value of the operator ${\hat{S}}_{z}$ (photon imbalance) as a function of *u* in the ground state of the Hamiltonian of Equation (3) for several values of the quantum number *S*: 10 (black), 15 (blue), 20 (red), 25 (green), 30 (magenta), and 40 (brown). For all curves, $g^{\prime }=1/2$, and $N_{\max}=5S$.

However, there are observables that are insensitive to the occurrence of degeneracy. One particularly interesting example is the imbalance operator ${\hat{S}}_{z}$ itself. The dependence of the mean value of this operator on the parameter λ is shown in Figure 3. As the value of *u* is increased, $\left|\langle {\hat{S}}_{z}\rangle \right|$ changes abruptly from zero to positive values for $u=u_{2}$, depending on the number of stored photons. After this abrupt change, $\left|\langle {\hat{S}}_{z}\rangle \right|/S$ tends to a value close to the unity, which means that the chosen ground state describes one of the two modes preferentially occupied. It is important to emphasize that such abrupt changes are not related to changes in the system, since they result from the diagonalization process. Nevertheless, they shed some light on some aspects of the two degenerate lowest energy eigenstates, as will be discussed below.

As discussed in Appendix B, the numerical diagonalization procedure of ${\hat{H}}^{\prime }$ can either pick up the eigenstate that describes *a*-mode preferentially populated, $\langle {\hat{S}}_{z}\rangle /S≃1$, or pick up that eigenstate describing *b*-mode populated, $\langle {\hat{S}}_{z}\rangle /S≃-1$. For each value of λ greater than $\lambda_{2}$, such a procedure can choose one or other eigenstate to represent $|\Psi_{0}\rangle$.

To suppress random abrupt jumps in the evaluation of the mean value of ${\hat{S}}_{z}$, a slight modification is implemented in the algorithm used to diagonalize ${\hat{H}}^{\prime }$. The two lowest energy degenerate eigenstates, $|\Psi_{0}\left(\lambda_{n}\right)\rangle$ and $|\Psi_{1}\left(\lambda_{n}\right)\rangle$ obtained for the current value of the control parameter, $\lambda_{n}$, are compared with the previous reference eigenstate $|\Psi_{r}\left(\lambda_{n-1}\right)\rangle$. As $\lambda_{n}$ is close to $\lambda_{n-1}$, one of the two lowest energy eigenstates, $|\Psi_{0}\left(\lambda_{n}\right)\rangle$ or $|\Psi_{1}\left(\lambda_{n}\right)\rangle$, has considerable overlap with $|\Psi_{r}\left(\lambda_{n-1}\right)\rangle$ and the other is almost orthogonal to it. Thus, the lowest energy state exhibing greatest overlap with the previous reference state will be chosen as the current reference eigenstate. Adopting this procedure, the mean value of the imbalance operator ${\hat{S}}_{z}$ as a function of λ is evaluated in the current ground state of ${\hat{H}}^{\prime }$ and the results are shown in Figure 3.

**Figure 4:**
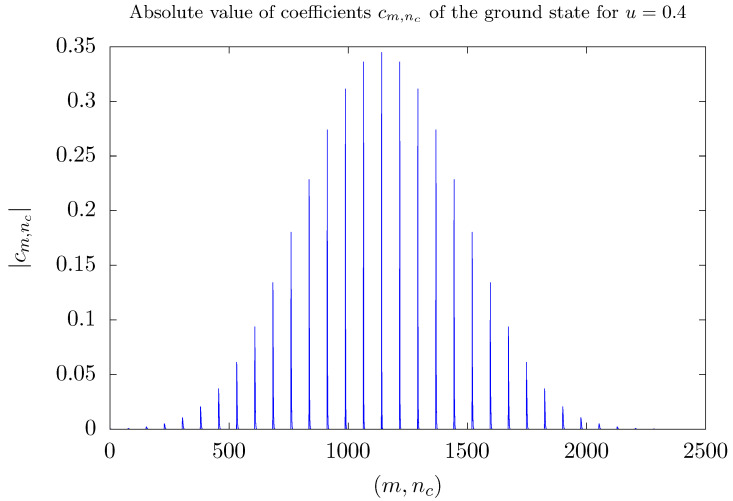
Modules of the coefficients $c_{m,n_{c}}$ of the ground state of ${\hat{H}}^{\prime }$ with respect to the computational basis ${\left\{|m,n_{c}\rangle \right\}}_{m=-S\ldots S,n_{c}=0\ldots N_{\max}}$ for u=0.4. Here, S=15 and $N_{\max}=5S$. States in the computational basis are ordered in ascending order as $|m=-15,n_{c}=0\rangle$, $|m=-15,n_{c}=1\rangle ,\ldots$, $|m=-15,n_{c}=75\rangle ,\ldots ,|m=15,n_{c}=75\rangle$. Numbers in the horizontal axis label states of the computational basis following this ordering. Variables are discrete and lines are guides to the eye.

**Figure 5:**
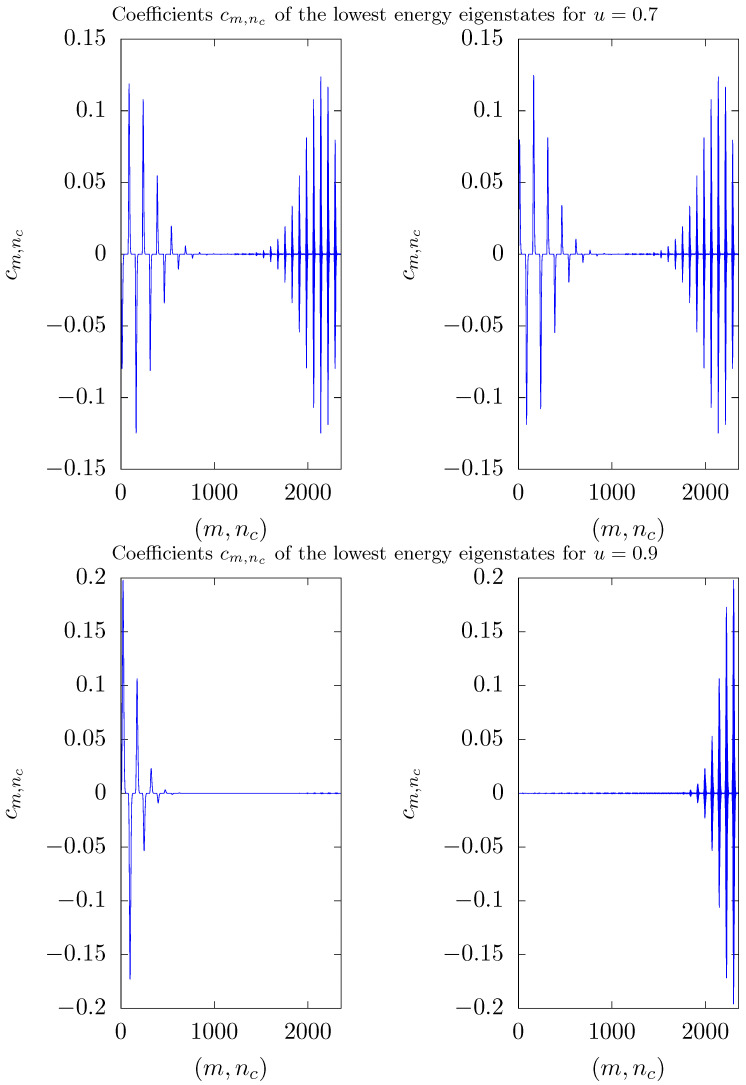
Coefficient distributions for the two lowest energy eigenstates of ${\hat{H}}^{\prime }$ with respect to the computational basis ${\left\{|m,n_{c}\rangle \right\}}_{m=-S\ldots S,n_{c}=0\ldots N_{\max}}$ for u=0.7 (top) and u=0.9 (bottom). Here, S=15 and $N_{\max}=5S$. States in the computational basis are ordered in ascending order as $|m=-15,n_{c}=0\rangle ,|m=-15,n_{c}=1\rangle ,\ldots ,|m=-15,n_{c}=75\rangle ,\ldots ,|m=15,n_{c}=75\rangle$. Numbers in the horizontal axis label states of the computational basis following this ordering. Variables are discrete and lines are guides to the eye.

The abrupt changes in curves of Figure 3 can be understood as a result of the diagonalization process. They occur for values of the coupling parameter $\lambda_{2}$ that depends on *S*, and is greater than the value $\lambda_{1}$, for which the two lowest energy eigenstates become degenerate, i.e., $\lambda_{2}>\lambda_{1}$. In this case, the two lowest energy eigenstates $|\Psi_{0}\rangle$ and $|\Psi_{1}\rangle$ define a two-dimensional eigensubspace, $W=\mathrm{span}\{|\Psi_{0}\rangle ,|\Psi_{1}\rangle \}$. Any state belonging to W can be picked up as the ground state by the diagonalization process. Therefore, the abrupt changes observed in curves of Figure 3 occur because, after the diagonalization process, a state that describes one of the two modes preferentially occupied is chosen in W. This can be better understood observing the distribution of the coefficients $c_{m,n_{c}}$ given by the expansion of the ground state in the computational basis. Figure 4 and Figure 5 show such a distribution for three different situations with S=15. To this end, states of the computational basis are arranged in an ascending order as follows: $|m=-15,n_{c}=0\rangle ,|m=-15,n_{c}=1\rangle ,\ldots ,|m=-15,n_{c}=N_{\max}\rangle ,\ldots ,|m=15,n_{c}=N_{\max}\rangle$. Hence, coefficients associated with negative values of *m* appear on the left side of the graphs, while coefficients assigned to positive values of *m* appear on the right side. Before degeneracy, as shown in Figure 4 for u=0.4, the coefficients are symmetrically distributed along the entire horizontal axis, exhibiting more significant values on the central part, where m∼0.

After degeneracy sets in, the distribution of the coefficients changes its shape. In fact, the coefficients vanish in the central part and are non-zero at the extremities of the graphs, as can be seen in Figure 5. This figure exhibits the coefficient distributions for both degenerate lowest energy eigenstates in two situations: after the degeneracy but before the abrupt change observed in Figure 3 (u=0.7) and after the abrupt change (u=0.9). In the first situation, for both eigenstates, non-vanishing coefficients appear on both extremities of the graph. However, for the other situation, with u=0.9, non-vanishing coefficients are concentrated at one extremity of the graph for one of the eigenstates, while for the other eigenstate, the coefficients are concentrated at the other extremity. Thus, for u=0.9, the eigenstate with coefficients distributed at the left extremity yields $\langle {\hat{S}}_{z}\rangle /S\approx -1$ whereas the eigenstate with coefficients distributed at the right extremity yields $\langle {\hat{S}}_{z}\rangle /S\approx 1$. In the situation where degeneracy has established but the abrupt change of $\langle {\hat{S}}_{z}\rangle$ has not yet occurred, the ground state describes a coherent superposition of these two eigenstates (a kind of Schrödinger’s cat state) that describes the photons preferentially occupying one of the modes.

We emphasize that the abrupt change observed in the curves of Figure 3 is not a result of any physical process, but rather it is a product of the diagonalization algorithm. For example, if an experimental realization of this system is performed in such way that the coupling parameter λ is adiabatically varied, it is unreasonable to expect this abrupt change to occur.

### 3.2. Fidelity Susceptibility

Fidelity between the pure states $|\phi \rangle$ and $|\psi \rangle$ measures how indistinguishable such states are. It can be measured by the overlap

$$F\left(\psi ,\phi \right)=\left|\langle \psi |\phi \rangle \right|.$$

As discussed above, for $\lambda <\lambda_{1}$, the ground state $|\Psi_{0}\rangle$ of the Hamiltonian $H^{\prime }$ describes both modes approximately equally populated, whilst for $\lambda >\lambda_{2}$, $|\Psi_{0}\rangle$ it describes one of the two modes preferentially populated. As the physical situations described by $|\Psi_{0}\rangle$ are very different for $\lambda <\lambda_{1}$ and for $\lambda >\lambda_{2}$, we expect a small overlap between the ground states immediately before and immediately after the onset of degeneracy and the occurrence of the abrupt change shown in Figure 3. In other words, considering ϵ positive and sufficiently small, we expect fidelity $F\left(\lambda -\epsilon ,\lambda +\epsilon \right)\equiv \left|\langle \Psi_{0}\left(\lambda -\epsilon \right)|\Psi_{0}\left(\lambda +\epsilon \right)\rangle \right|$ suffering a significant decay for $\lambda =\lambda_{1}$ and $\lambda =\lambda_{2}$ if compared with other values of λ.

For our purposes, instead of using direct fidelity, we opt to work with an inherited measure — the fidelity susceptibility [27,50,51,52,53,54,55,56]. Defining the fidelity between the ground states $|\Psi_{0}\left(\lambda \right)\rangle$ and $|\Psi_{0}\left(\lambda +\delta \right)\rangle$ as $F\left(\lambda ,\delta \right)$, the fidelity susceptibility is given by

(7) $$\chi_{F}\left(\lambda \right)\equiv -\frac{1}{2}{\left.\frac{\partial^{2}F\left(\lambda ,\delta \right)}{\partial \delta^{2}}\right|}_{\delta =0}.$$

**Figure 6:**
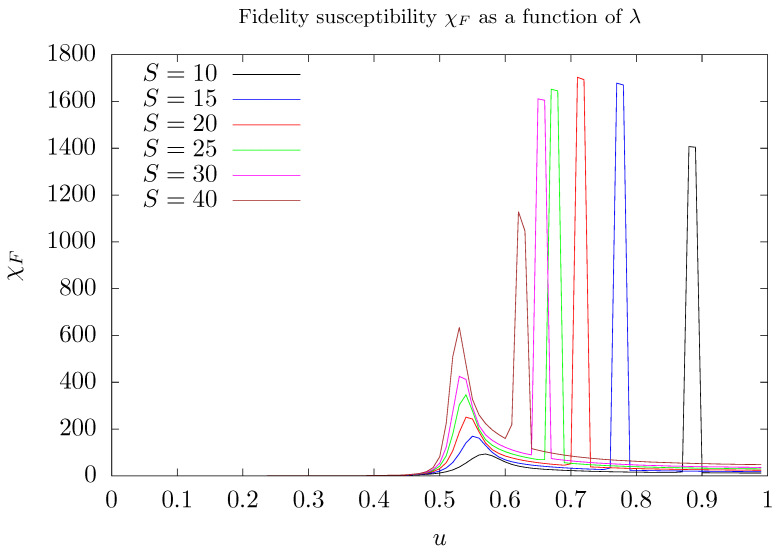
Fidelity susceptibility $\chi_{F}$ between ground states of the Hamiltonian of Equation (3) as a function of the parameter λ (*u*) for several values of *S*. We use $N_{\max}=5S$ as truncation of the maximum number of phonons considered in our simulations. Here, $\lambda =u/\sqrt{S}$. Different colors were attributed to different values of *S*: 10 (black), 15 (blue), 20 (red), 25 (green), 30 (magenta), and 40 (brown).

Figure 6 shows the results of numerical evaluation of fidelity susceptibility as a function of the parameter λ for several values of the quantum number *S*. For values of $\lambda <\lambda_{1}$, $\chi_{F}$ is approximately constant and close to zero. As λ increases, but still keeping smaller than the critical value, a bump arises around u=1/2, signalizing the appearance of degeneracy of the two lowest energy eigenstates. At the critical value $\lambda_{2}$, $\chi_{F}$ exhibits a high peak that quickly drops and, for $\lambda >\lambda_{2}$, becomes approximately constant. As the quantum number *S* increases, these peaks seem to accumulate around a given value of the parameter λ — the critical value $\lambda_{1}$ corresponding to the appearance of degeneracy. The peak signalizes that the two lowest energy and degenerate eigenstates now describe photons stored in one mode and the other empty instead of describing a quantum superposition of such eigenstates.

### 3.3. Fluctuations

Order parameters are quantities selected to distinguish two different phases of a given macroscopic system and, to be measurable, fluctuations of these quantities must be mild compared with their measured values when the system is found in any of its phases [31,57]. At the transition point, it is expected that such fluctuations diverge. A question that naturally arises is how fluctuations behave when a finite system undergoes changes similar to those applied to the corresponding macroscopic system during a phase transition.

**Figure 7:**
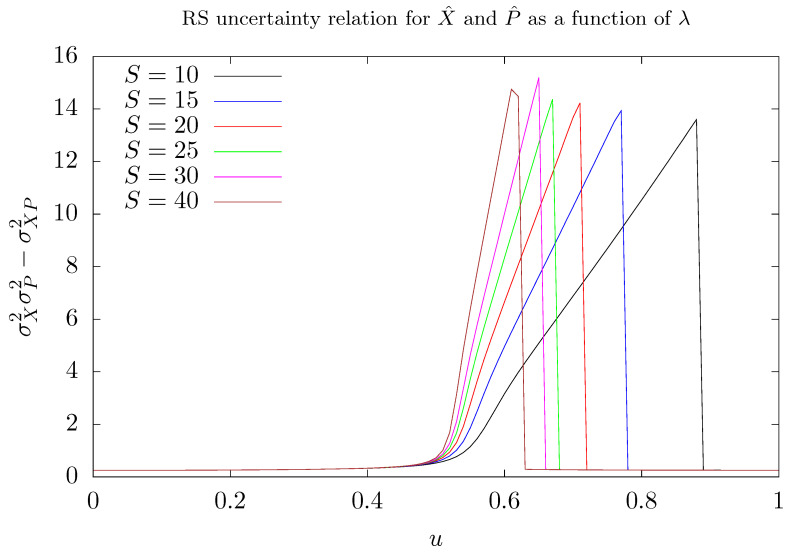
Fluctuations of membrane’s observables $\hat{X}$ and $\hat{P}$ measured by RS uncertainty relation as a function of the parameter λ (*u*) in the ground state of the Hamiltonian ${\hat{H}}^{\prime }$, for several values of *S*. We use $N_{\max}=5S$ as truncation of the maximum number of phonons considered in our simulations. Here, $\lambda =u/\sqrt{S}$. Different colors were attributed to different values of *S*: 10 (black), 15 (blue), 20 (red), 25 (green), 30 (magenta), and 40 (brown).

In order to shed some light on the role of fluctuations on the model studied here, we evaluate the Robertson-Schrödinger (RS) uncertainty relation [58,59] for the membrane’s observables $\hat{X}$ and $\hat{P}$ as the coupling parameter λ is varied for different values of *S*. In the Dicke model, fluctuations have been used as an indicator of chaos [60] and of quantum phase transition in the mean field approximation [61]. The variances of these observables were evaluated in the ground state of the Hamiltonian ${\hat{H}}^{\prime }$. The results are presented in Figure 7. RS uncertainty is given by

(8) $$\sigma_{X}^{2}\sigma_{P}^{2}-\sigma_{XP}^{2}\geq \frac{1}{4},$$

where $\sigma_{X}^{2}=\langle {\hat{X}}^{2}\rangle -{\langle \hat{X}\rangle }^{2}$ and $\sigma_{P}^{2}=\langle {\hat{P}}^{2}\rangle -{\langle \hat{P}\rangle }^{2}$ stand for the variances of $\hat{X}$ and $\hat{P}$ operators, whilst $\sigma_{XP}=\frac{1}{2}\langle \hat{X}\hat{P}+\hat{P}\hat{X}\rangle -\langle \hat{X}\rangle \langle \hat{P}\rangle$ stands for the corresponding covariance. According to Figure 7, if λ is lower than $\lambda_{1}$ or greater than $\lambda_{2}$, then fluctuations are relatively small. However, at the critical value $\lambda_{1}$, when degeneracy appears, fluctuations start to increase quasi-linearly until λ reaches the value $\lambda_{2}$, corresponding to a situation in which the ground state describes one of the modes preferentially populated. When this value is reached, fluctuations quickly drop and remain constant after that. Note that, as the quantum number *S* increases, i.e., as the number of stored photons increases, fluctuations grow more rapidly with λ.

The ground state is kind of a Schrödinger’s cat state when λ runs in the range $\left[\lambda_{1},\lambda_{2}\right]$ and, due to the delocalization, the position-momentum uncertainty relation increases. In this situation, the ground state is a coherent superposition of states describing photons trapped in one mode and the membrane displaced from its rest position as a result of the radiation pressure. As a byproduct of the diagonalization process, when $\lambda >\lambda_{2}$, the ground state is now a localized state, as if the expected superposition had collapsed into one of the states that form it.

### 3.4. Mean Force on the Membrane

Establishing an analogy with classical physics, let us define a “force on membrane” operator as the corresponding time derivative of the membrane linear momentum. From the Hamiltonian given by Equation (1), we have

$$\begin{array}{ccc} \frac{d}{dt}\hat{p} & = & -\frac{1}{i\hbar }\left[\hat{p},\hat{H}\right]\\ & = & -m\omega^{2}\hat{x}-\frac{2}{L}\hbar \omega_{0}\left({\hat{n}}_{a}-{\hat{n}}_{b}\right), \end{array}$$

or, in terms of dimensionless operators and parameters,

(9) $$\frac{d}{d\tau }\hat{P}=-\hat{X}-\lambda \left({\hat{n}}_{a}-{\hat{n}}_{b}\right)=-\frac{1}{\sqrt{2}}\left(\hat{c}+{\hat{c}}^{†}\right)-2\lambda {\hat{S}}_{z},$$

where τ=ωt is a dimensionless time. Let us define the dimensionless force operator as $\hat{F}=\frac{d}{d\tau }\hat{P}$. We recognize two contributions to $\hat{F}$ in Equation (9): the first term represents the elastic restoring force (we called it harmonic contribution); the second term is due to the radiation pressure acting on the membrane and it is proportional to the photon imbalance of the *a* and *b* modes. To analyze how the force on the membrane is affected as the parameter λ varies, we evaluated its mean value in the ground state of the Hamiltonian given by Equation (3). As expected, the two different contributions to $\hat{F}$ are opposite.

**Figure 8:**
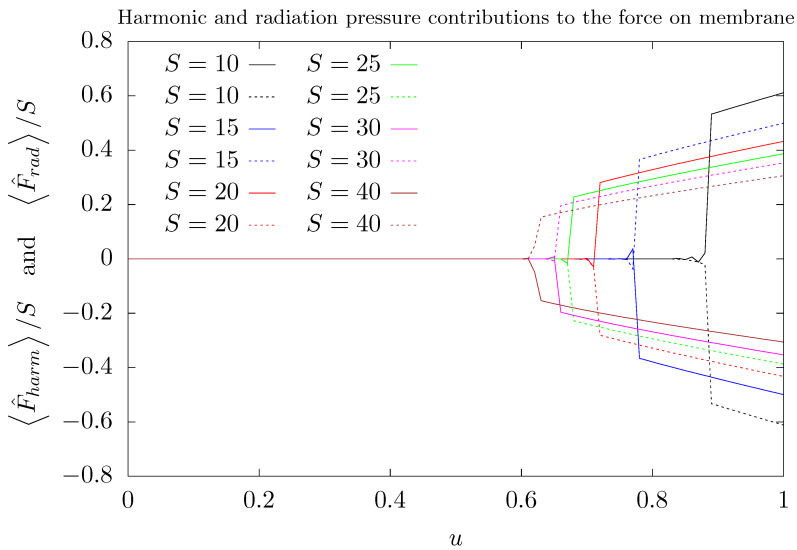
Mean values of the two contributions to the force on the membrane $\hat{F}$ as functions of the parameter λ (*u*) for several values of *S* in the ground state of the Hamiltonian of Equation (3): ${\hat{F}}_{\mathrm{harm}}$ (harmonic contribution—continuous line) and ${\hat{F}}_{\mathrm{rad}}$ (radiation pressure contribution—dashed line). We use $N_{\max}=5S$ as truncation of the maximum number of phonons considered in our simulations. Here, $\lambda =u/\sqrt{S}$. Different colors were attributed to different values of *S*: 10 (black), 15 (blue), 20 (red), 25 (green), 30 (magenta), and 40 (brown).

Figure 8 shows the mean value of these two contributions, $\langle {\hat{F}}_{\mathrm{harm}}\rangle =-\langle \hat{X}\rangle$ and $\langle {\hat{F}}_{\mathrm{rad}}\rangle =-\lambda \langle {\hat{n}}_{a}-{\hat{n}}_{b}\rangle$, as a function of λ. For $\lambda <\lambda_{2}$, the mean values of these contributions are close to zero and, when the value $\lambda_{2}$ is reached, both suffer an abrupt jump. The mean values of the harmonic and radiation pressure contributions linearly grow with λ for $\lambda >\lambda_{2}$, and they keep the same absolute magnitude, but opposite signs. Thus, the restoring harmonic force equilibrates the force applied on the membrane by the radiation pressure resulting from the imbalance in the photon population of the two modes. As a consequence, for any value of the coupling parameter λ, the mean value of the force on membrane is null. For $\lambda >\lambda_{2}$, for each value of λ, in the chosen ground state, the membrane reaches an equilibrium position, given by

(10) $$\langle \hat{X}\rangle =-\lambda \langle {\hat{n}}_{a}-{\hat{n}}_{b}\rangle \approx \pm 2\lambda S.$$

The signal “±” in the right hand side of the above equation is due to the degeneracy of the two lowest energy eigenstates. As discussed, each one of these eigenstates describes one of the two modes populated for $\lambda >\lambda_{2}$.

## 4. Conclusions

In this work, we studied a two-mode optomechanical model that is equivalent to the well-known *N*-atom Dicke model of quantum optics. The Dicke model has a richness of phenomena, such as QPT and its classical limit can exhibits chaos. Using such an equivalence, we analyze the behavior of mean values of three chosen observables with respect to the parameter λ that measures the coupling between the membrane and the light modes in the ground state of the Hamiltonian (3).

The numerical results presented in previous section show the existence of two different values of the coupling parameter λ for which the two-mode optomechanical system undergoes significant changes. For $\lambda =\lambda_{1}\approx 1/\left(2\sqrt{S}\right)$, the two lowest energy eigenstates of the Hamiltonian ${\hat{H}}^{\prime }$ become degenerate. For a second value $\lambda_{2}>\lambda_{1}$, the ground state describes one of the two cavity modes preferentially populated. In fact, they are a product of the diagonalization process of the Hamiltonian ${\hat{H}}^{\prime }$. After the onset of degeneracy, for λ in $\left[\lambda_{1},\lambda_{2}\right]$, the algorithm used to diagonalize ${\hat{H}}^{\prime }$ chooses ground states that are characterized as coherent superpositions of eigenstates that describe one of the two modes preferentially occupied. Let such eigenstates be identified as $|\Psi^{+}\rangle$ and $|\Psi^{-}\rangle$, with each of them describing photons populating one of the modes, i.e., $\langle \Psi^{\pm }|{\hat{S}}_{z}|\Psi^{\pm }\rangle \approx \pm S$ and $\langle \Psi^{\pm }|\Psi^{\mp }\rangle =0$. Soon after the degeneracy, the two lowest energy eingenstates are coherent superpositions of $|\Psi^{+}\rangle$ and $|\Psi^{-}\rangle$. Nevertheless, for $\lambda >\lambda_{2}$, the roles of these lowest energy eigenstates are played by $|\Psi^{+}\rangle$ and $|\Psi^{-}\rangle$ themselves. The value of $\lambda_{2}$ depends on the quantum number *S*, or in other words, on the total number of photons stored in the cavity.

**Figure A2:**
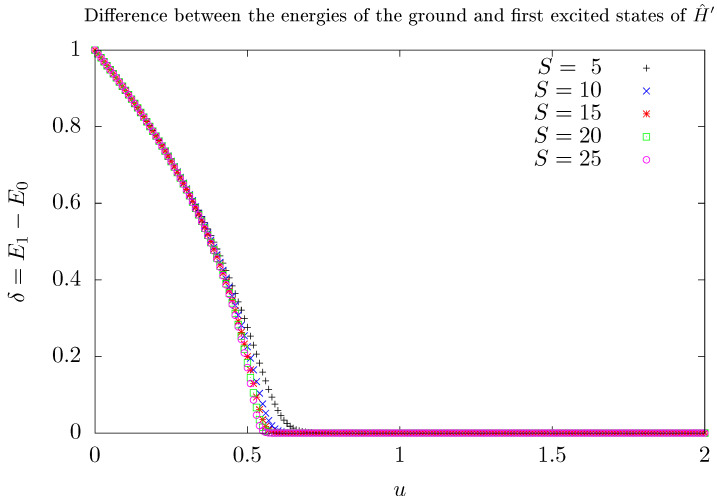
Energy separation of ground and first excited states with respect to λ (*u*), $\delta \left(\lambda \right)=E_{1}\left(\lambda \right)-E_{0}\left(\lambda \right)$, for different values of *S*. Note that the energy separation is zero for values of λ greater than a critical value. For all curves, $g^{\prime }=1/2$, $N_{\max}=15S$ and $\lambda =u/\sqrt{S}$. Different colors and symbols were attributed to different values of *S*: 5 (black plus), 10 (blue cross), 15 (red asterisk), 20 (green square), and 25 (magenta circle).

As shown in Figure 6, as the parameter λ varies, fidelity susceptibility $\chi_{F}$ curve exhibits a bump followed by a high peak that quickly drops. The bump announces the settlement of degeneracy between the two lowest energy eigenstates of ${\hat{H}}^{\prime }$, whilst the peak signalizes that the ground state describes the trapping of photons in one of the two modes, a numerical effect, as pointed out above. Note that, as the number of stored photons increases, the distance between the bump and the peak diminishes. Such a behavior is confirmed by the numerical calculations of $\langle {\hat{S}}_{z}\rangle \left(\lambda \right)$ and $\chi_{F}\left(\lambda \right)$. The settlement of degeneracy can be verified in Figure A2 in Appendix B which shows the energy separation δ of the two lowest energy eigenstates as a function of λ. Despite how imbalance ${\hat{S}}_{z}$ is insensitive to the emergence of degeneracy, imbalance squared is sensitive, as can be verified in Figure 2.

Finally, we were able to verify how the force on the membrane $\hat{F}$ is affected close to $\lambda_{2}$ for different values of *S*. $\hat{F}$ has two opposite contributions, analogous to a Newtonian pair of action-reaction forces: one of them proportional to the membrane position $\hat{X}$ (related to the harmonic restoring force) and the other proportional to the photon imbalance ${\hat{S}}_{z}=\frac{1}{2}\left({\hat{n}}_{a}-{\hat{n}}_{b}\right)$ (assigned to the radiation pressure). For lower values of λ, both contributions to $\langle \hat{F}\rangle$ are close to zero, because both modes are equally occupied. At $\lambda =\lambda_{2}$, both contributions jumps to non-null opposite values with the same absolute magnitude. After the transition, in the ground state, both contributions linearly grow with λ, keeping the same absolute magnitude but with opposite signals. Thus, the mean value of $\hat{F}$ is kept null, regardless of the value of λ. However, in this situation, as a reaction of the restoring force to the radiation pressure, the membrane reaches a new equilibrium position that depends on the total number of photons stored in the cavity. Resembling the Newton’s third law of motion, even after the the photon trapping in one mode, the mean value of the net force on the membrane in the ground state is null, and as a reaction to the force due to the radiation pressure, the membrane reaches a new equilibrium position that depends on the number of photons stored in the cavity. This behavior can be considered as a finite-size correspondent of the buckling phase transition in symmetric optomechanical cavities, for which a recent experimental test was recently reported in Ref. [62].

As emphasized throughout this work, we consider the closed system approximation in our model that may make its experimental test difficult, considering the current state-of-art of experiments involving optomechanical devices. A test of this model could be carried out on a device similar to that described in Ref. [62]. However, a superconducting cavity could be needed to increase the lifetime of photons stored there. Therefore, such a device would require cryogenic and vacuum systems. Furthermore, cryogenics would be necessary to reduce the presence of thermal excitations in both the cavity and the membrane.

## Figures and Tables

**Figure A1:**
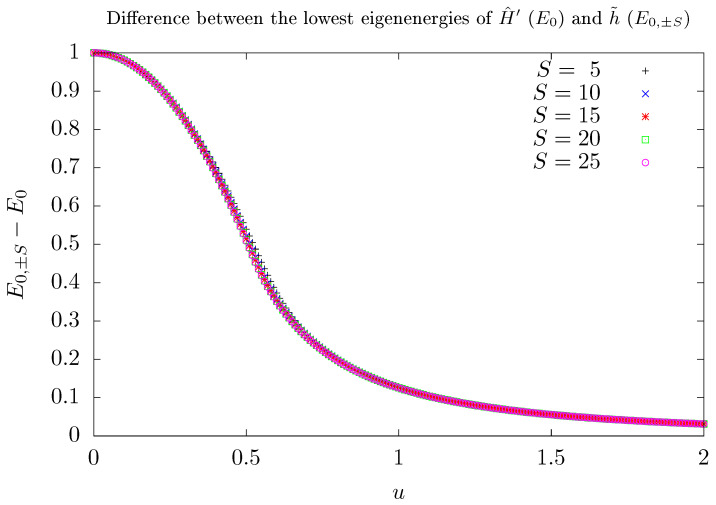
Difference between the energies of the ground states of ${\hat{H}}^{\prime }$ and $\tilde{h}$. We use $N_{\max}=15S$ as truncation of the maximum number of phonons considered in our simulations. Here, $\lambda =u/\sqrt{S}$. Different colors and symbols were attributed to different values of *S*: 5 (black plus), 10 (blue cross), 15 (red asterisk), 20 (green square), and 25 (magenta circle).

## Data Availability

Codes (in Python) and data used in this work can be downloaded by accessing https://osf.io/y9ust/?view_only=af4bec4f9e4b48e29f5aa5e708abaef6 (accessed on 1 October 2023).

## Appendix A. Calculation of the Critical Value *λ*_1_

Changes in the structure of energy levels of a quantum Hamiltonian can be signalized by changes in the structure of the corresponding classical phase space [63,64]. As we discussed above, when the coupling parameter λ reaches first critical value $\lambda_{1}$, the optomechanical model exhibits degeneracy. To calculate the value of $\lambda_{1}$, we resort to the thermodynamic limit of the Hamiltonian given Equation (3). Defining $\hat{H}\equiv {\hat{H}}^{\prime }/S$, we obtain

(A1) $$\hat{H}={\hat{\gamma }}^{†}\hat{\gamma }+2g^{\prime }{\hat{s}}_{x}+\sqrt{2}u\left({\hat{\gamma }}^{†}+\hat{\gamma }\right){\hat{s}}_{z},$$

where $\hat{\gamma }\equiv \hat{c}/\sqrt{S}$, and ${\hat{s}}_{i}\equiv {\hat{S}}_{i}/S$, for i=x,y,z, are the new operators, and $u\equiv \lambda \sqrt{S}$ is the renormalized coupling parameter. Note that, in the limit of S→∞, which is equivalent to the number of stored photons in the cavity going to infinity, the commutation relations are given by

(A2) $$\begin{array}{cc} \lim_{S\to \infty }\left[{\hat{\gamma }}^{†},\hat{\gamma }\right] & =\lim_{S\to \infty }\frac{1}{S}\left[{\hat{c}}^{†},\hat{c}\right]=\lim_{S\to \infty }\frac{1}{S}=0, \end{array}$$

(A3) $$\begin{array}{cc} \lim_{S\to \infty }\left[{\hat{s}}_{i},{\hat{s}}_{j}\right] & =\lim_{S\to \infty }\frac{1}{S^{2}}\left[{\hat{S}}_{i},{\hat{S}}_{j}\right]=\lim_{S\to \infty }\frac{1}{S^{2}}\varepsilon_{ijk}{\hat{S}}_{k}=0, \end{array}$$

where $\varepsilon_{ijk}$ stands for the antisymmetric Levi-Civita symbol. In the limit S→∞, operators in the Hamiltonian (A1) can be substituted by c-number functions giving origin to the classical Hamiltonian

(A4) $$H=\lim_{S\to \infty }\hat{H}=\gamma^{*}\gamma +2g^{\prime }\sqrt{1-s_{z}^{2}}\cos\phi +\sqrt{2}u\left(\gamma^{*}+\gamma \right)s_{z}.$$

Here, $\gamma =\lim_{S\to \infty }\hat{\gamma }$, $\gamma^{*}=\lim_{S\to \infty }{\hat{\gamma }}^{†}$, $s_{z}=\lim_{S\to \infty }{\hat{S}}_{z}$, with $-1\leq s_{z}\leq 1$, and ϕ, with 0≤ϕ≤π, are the dynamical variables of this classical Hamiltonian. To keep the renormalized coupling parameter *u* finite as *S* goes to infinite, λ goes to zero.

The classical Hamiltonian in Equation (A4) depends on four dynamical variables. The classical phase space is embedded in a four-dimensional space, so as the parameters vary, the structure of the orbits along with the nature of the critical points also change. The critical points of the Hamiltonian H can be evaluated by

(A5) $$\begin{array}{cc} \frac{\partial H}{\partial \gamma } & =\gamma^{*}+\sqrt{2}us_{z}, \end{array}$$

(A6) $$\begin{array}{cc} \frac{\partial H}{\partial \gamma^{*}} & =\gamma +\sqrt{2}us_{z}, \end{array}$$

(A7) $$\begin{array}{cc} \frac{\partial H}{\partial s_{z}} & =-\frac{2g^{\prime }s_{z}\cos\phi }{\sqrt{1-s_{z}^{2}}}+\sqrt{2}u\left(\gamma^{*}+\gamma \right) \end{array}$$

(A8) $$\begin{array}{cc} \frac{\partial H}{\partial \phi } & =-2g^{\prime }\sqrt{1-s_{z}^{2}}\sin\phi . \end{array}$$

Making null or undefined the derivatives in Equations (A5)–(A8), we obtain the following set of critical points:

(A9) $$\begin{array}{cc} \gamma =\gamma^{*} & =0,\;s_{z}=0,\;\phi =0\;\mathrm{or}\;\pi , \end{array}$$

(A10) $$\begin{array}{cc} \gamma =\gamma^{*} & =\pm u\sqrt{2\left(1-\frac{g^{\prime 2}}{4u^{4}}\right)},\;s_{z}=\mp \sqrt{1-\frac{g^{\prime 2}}{4u^{4}}},\;\phi =0\;\mathrm{or}\;\pi ,\;\mathrm{and} \end{array}$$

(A11) $$\begin{array}{cc} \gamma =\gamma^{*} & =\pm \sqrt{2}u,\;s_{z}=\mp 1,\;\mathrm{and}\;\phi \;\mathrm{is}\;\mathrm{undefined}. \end{array}$$

In our simulations, we made $g^{\prime }=1/2$. Thus, assuming $s_{z}$ real, the critical point in Equation (A10) should only be considered for u≥1/2. For u=1/2, Equations (A9) and (A10) represents the same critical point, and as *u* takes values greater than 1/2, such critical points separate.

The Hessian matrix corresponding to the Hamiltonian H is

(A12) $$H\left(\gamma ,\gamma^{*},s_{z},\phi \right)=\left[\begin{array}{cccc} 1 & 0 & \sqrt{2}u & 0\\ 0 & 1 & \sqrt{2}u & 0\\ \sqrt{2}u & \sqrt{2}u & -\frac{2g^{\prime }\cos\phi }{{\left(1-s_{z}^{2}\right)}^{3/2}} & \frac{2g^{\prime }s_{z}\sin\phi }{\sqrt{1-s_{z}^{2}}}\\ 0 & 0 & \frac{2g^{\prime }s_{z}\sin\phi }{\sqrt{1-s_{z}^{2}}} & -2g^{\prime }\sqrt{1-s_{z}^{2}}\cos\phi \end{array}\right].$$

Equation (A9) provides two critical points, one for ϕ=0 and the other for ϕ=π. For $g^{\prime }=1/2$, we have $\det H\left(0,0,0,0\right)=1+4u^{2}$, that is always positive whatever the value of *u*. In this case, Hessian matrix H is not positive defined nor negative defined, and the critical point in Equation (A9) for ϕ=0 corresponds to a saddle point. However, $\det H\left(0,0,0,\pi \right)=1-4u^{2}$, that is positive for 0≤u<1/2 and negative for u>1/2. Note that the nature of this last critical point changes if u=1/2. In fact, for 0≤u<1/2, H is positive defined (local minimum) but, for u>1/2, positiveness/negativeness of H is not defined (saddle point). For the case of Equation (A10), we define $\tilde{\gamma }=u\sqrt{2\left(1-\frac{g^{\prime 2}}{4u^{4}}\right)}$ and ${\tilde{s}}_{z}=\sqrt{1-\frac{g^{\prime 2}}{4u^{4}}}$. This equation provides four critical points depending on the value taken for ϕ and the signal chosen for γ and $s_{z}$. Thus, for $g^{\prime }=1/2$, we have

$$\det H\left(\pm \tilde{\gamma },\pm {\tilde{\gamma }}^{*},\mp {\tilde{s}}_{z},\pi \right)=8u^{4}-1/2$$

and

$$\det H\left(\pm \tilde{\gamma },\pm {\tilde{\gamma }}^{*},\mp {\tilde{s}}_{z},0\right)=8u^{4}+1/2$$

As mentioned above, these critical points are meaningless if u<1/2. For u>1/2, the critical points in Equation (A10) for ϕ=0 are saddle points, since positiveness/negativeness of H is not defined. However, for ϕ=π and for u>1/2, the critical points in Equation (A10) are local minima, by virtue of the positiveness of H. In short, the critical point $\gamma =\gamma^{*}=0,\;s_{z}=0,\;\phi =\pi$ is a local minimum for 0<u<1/2 and turns into a saddle point for u>1/2, and two new local minima simmetrically localized around it appear at $\gamma =\gamma^{*}=\pm u\sqrt{2\left(1-\frac{g^{\prime 2}}{4u^{4}}\right)},\;s_{z}=\mp \sqrt{1-\frac{g^{\prime 2}}{4u^{4}}},\;\phi =\pi$. Again, the nature of the critical points related with ϕ=π is changed when *u* reaches the value 1/2. In our simulations, degeneracy occurs for u≈1/2, in agreement with the critical value found using the classical version of the Hamiltonian ${\hat{H}}^{\prime }$.

## Appendix B. Some Aspects of Diagonalization of the Two-Mode Optomechanical Model Hamiltonian

We start with the Hamiltonian that describes the interaction of the two-mode of the electromagnetic field with a membrane, given by Equation (3). Introducing the generalized displacement operator

(A13) $$\hat{D}\left(\hat{\xi }\right)=\exp\left(\hat{\xi }{\hat{c}}^{†}-{\hat{\xi }}^{†}\hat{c}\right),$$

that, when acting on $\hat{c}$ and ${\hat{c}}^{†}$, yields [65]

(A14) $$\hat{D}\left(\hat{\xi }\right)\hat{c}{\hat{D}}^{†}\left(\hat{\xi }\right)=\hat{c}-\hat{\xi },\;\;\mathrm{and}\;\;\hat{D}\left(\hat{\xi }\right){\hat{c}}^{†}{\hat{D}}^{†}\left(\hat{\xi }\right)={\hat{c}}^{†}-{\hat{\xi }}^{†},$$

where

(A15) $$\hat{\xi }=\alpha {\hat{S}}_{z}.$$

$\hat{D}\left(\hat{\xi }\right)$ is unitary, since ${\hat{D}}^{†}\left(\hat{\xi }\right)={\hat{D}}^{-1}\left(\hat{\xi }\right)=\hat{D}\left(-\hat{\xi }\right)$. Now, consider the Hamiltonian

(A16) $$\hat{h}={\hat{n}}_{c}-2\lambda^{2}{\hat{S}}_{z}^{2}.$$

$\left\{|m,n_{c}\rangle \equiv |m\rangle ⊗|n_{c}\rangle \right\}$, with $n_{c}=0,1,\ldots$, and −S≤m≤S, is the set of eigenstates of $\hat{h}$ with eigenvalues $E_{n_{c},m}=n_{c}-2\lambda^{2}m^{2}$. Making $\alpha =\sqrt{2}\lambda$ in (A15), we define

(A17) $$\tilde{h}={\hat{D}}^{†}\left(\hat{\xi }\right)\hat{h}\hat{D}\left(\hat{\xi }\right)=\left({\hat{c}}^{†}+\sqrt{2}\lambda {\hat{S}}_{z}\right)\left(\hat{c}+\sqrt{2}\lambda {\hat{S}}_{z}\right)-2\lambda^{2}{\hat{S}}_{z}^{2}={\hat{n}}_{c}+\sqrt{2}\lambda {\hat{S}}_{z}\left(\hat{c}+{\hat{c}}^{†}\right).$$

Note that $\tilde{h}={\hat{H}}^{\prime }-2g^{\prime }{\hat{S}}_{x}$. Moreover, the eigenstates of $\tilde{h}$ form the set $\left\{|m;-\xi_{m},n_{c}\rangle \equiv |m\rangle ⊗|-\xi_{m},n_{c}\rangle \right\}$, where $|-\xi_{m},n_{c}\rangle \equiv {\hat{D}}^{†}\left(\xi_{m}\right)|n_{c}\rangle$, with $\xi_{m}=\sqrt{2}\lambda m$, is a displaced number state [66,67]. The respective eigenvalues are

(A18) $$E_{n_{c},m}=n_{c}-2\lambda^{2}m^{2}=n_{c}-\xi_{m}^{2}.$$

Note that the eigenstates with m≠0 are at least twofold degenerate. In fact, for a given $n_{c}$, the states $|m;-\xi_{m},n_{c}\rangle$ and $|-m;-\xi_{-m},n_{c}\rangle$, m≠0, have the same energy $E_{n_{c},m}=E_{n_{c},-m}$. In particular, the ground state of $\tilde{h}$ is twofold degenerate with energy $E_{0,-S}=E_{0,S}=-2\lambda^{2}S^{2}$. To illustrate the convergence of the lowest eigenenergy of ${\hat{H}}^{\prime }$ to the corresponding one of $\tilde{h}$ as λ grows, Figure A1 shows the difference between them. As $n_{c}=0$, the ground state of $\tilde{h}$ is $|\pm S,\pm \sqrt{2}\lambda S\rangle =|\pm S\rangle ⊗|\pm \sqrt{2}\lambda S\rangle$, where $|\pm \sqrt{2}\lambda S\rangle$ stands for the coherent state $\hat{D}\left(\pm \sqrt{2}\lambda S\right)|0\rangle$.

In this work, we chose the ordinary phonon number states $|n_{c}\rangle$ to diagonalize the Hamiltonian ${\hat{H}}^{\prime }$. As the dimension of the Hilbert space associated to the mechanical subsystem is infinite, some truncation must be adopted for the quantum number $n_{c}$ in the numerical diagonalization of ${\hat{H}}^{\prime }$. Assume

(A19) $$|\psi \rangle =\sum_{m=-S}^{S}\sum_{n_{c}=0}^{N_{\max}}c_{m,n_{c}}|m,n_{c}\rangle$$

as an unknown eigenvector of ${\hat{H}}^{\prime }$ associated to an unknown eigenvalue ϵ, ${\hat{H}}^{\prime }|\psi \rangle =\epsilon |\psi \rangle$. The range of the quantum number $n_{c}$ was truncated by $N_{\max}$, i.e., $0\leq n_{c}\leq N_{\max}$. There are $\left(2S+1\right)\left(N_{\max}+1\right)$ unknown coefficients $\left\{c_{m,n_{c}}\right\}$ to determine, alongside ϵ itself. These unknowns are numerically obtained by solving a set of linear equations represented by

(A20) $$\sum_{m=-S}^{S}\sum_{n_{c}=0}^{N_{\max}}H_{m^{\prime },n_{c}^{\prime };m,n_{c}}^{\prime }c_{m,n_{c}}=\epsilon c_{m^{\prime },n_{c}^{\prime }}.$$

Naturally, the choice of phonon number truncation $N_{\max}$ affects the computation time used in the numerical diagonalization. Moreover, this choice affects the quality of the evaluation of mean values of observables in the ground state with respect to the coupling parameter λ (or its equivalent *u*). If the targeted observable acts on the two-mode subsystem space, the respective mean value converges quickly with $N_{\max}$, as it happens with the photon imbalance ${\hat{S}}_{z}$. However, the convergence is slow if the targeted observable acts on the membrane subsystem space. In other words, the value chosen for $N_{\max}$ limits the range of variation for *u*. In our simulations, we adopt $N_{\max}=5S$, which has been shown suitable to preserve the convergence of mean values of observables as *u* varies in the interval $\left[0,1\right]$.

Another point to be considered when diagonalizing the Hamiltonian ${\hat{H}}^{\prime }$ is the appearance of degenerate states for sufficiently large values of λ/g. To confirm the presence of the degeneracy, we evaluated the energy separation of ground and first excited states with respect to λ, $\delta \left(\lambda \right)=E_{1}\left(\lambda \right)-E_{0}\left(\lambda \right)$ for different values of *S*. In Figure A2, we plot curves of $\delta \left(\lambda \right)$ for different values of *S*. For a fixed *S*, the energy separation goes to zero as λ is increased, reaching zero at $\lambda_{1}$, and is kept equal to zero if $\lambda >\lambda_{1}$. The degeneracy affects the “choice” of one of the degenerated states as the ground state by our numerical treatment. In fact, if degeneracy is settled, the algorithm can choose any state living in a two-dimensional eigensubspace as a ground state. Under the degeneracy and for larger values of λ, the algorithm chooses states that describe photons occupying one of the modes. For moderate values, the ground state is a coherent superposition of these last ones.

## References

[1] Hollander E., Gottlieb O. (2021). Global bifurcations and homoclinic chaos in nonlinear panel optomechanical resonators under combined thermal and radiation stresses. Nonlinear Dyn..

[2] Bowen W.P., Milburn G.J. (2016). Quantum Optomechanics.

[3] Kippenberg T.J., Vahala K.J. (2008). Cavity optomechanics: Back-action at the mesoscale. Science.

[4] Aspelmeyer M., Meystre P., Schwab K. (2012). Quantum Optomechanics. Phys. Today.

[5] Braginsky V., Vyatchanin S. (2002). Low quantum noise tranquilizer for Fabry–Perot interferometer. Phys. Lett. A.

[6] Clerk A.A., Marquardt F., Jacobs K. (2008). Back-action evasion and squeezing of a mechanical resonator using a cavity detector. New J. Phys..

[7] Hertzberg J.B., Rocheleau T., Ndukum T., Savva M., Clerk A.A., Schwab K.C. (2010). Back-action-evading measurements of nanomechanical motion. Nat. Phys..

[8] Børkje K. (2020). Critical quantum fluctuations and photon antibunching in optomechanical systems with large single-photon cooperativity. Phys. Rev. A.

[9] Bhattacharya M., Meystre P. (2007). Trapping and cooling a mirror to its quantum mechanical ground state. Phys. Rev. Lett..

[10] Bhattacharya M., Uys H., Meystre P. (2008). Optomechanical trapping and cooling of partially reflective mirrors. Phys. Rev. A.

[11] Teufel J.D., Donner T., Li D., Harlow J.W., Allman M.S., Cicak K., Sirois A.J., Whittaker J.D., Lehnert K.W., Simmonds R.W. (2011). Sideband cooling of micromechanical motion to the quantum ground state. Nature.

[12] Chan J., Alegre T.P.M., Safavi-Naeini A.H., Hill J.T., Krause A., Gröblacher S., Aspelmeyer M., Painter O. (2011). Laser cooling of a nanomechanical oscillator into its quantum ground state. Nature.

[13] Weis S., Rivière R., Deléglise S., Gavartin E., Arcizet O., Schliesser A., Kippenberg T.J. (2010). Optomechanically induced transparency. Science.

[14] Marshall W., Simon C., Penrose R., Bouwmeester D. (2003). Towards quantum superpositions of a mirror. Phys. Rev. Lett..

[15] Kleckner D., Pikovski I., Jeffrey E., Ament L., Eliel E., Van Den Brink J., Bouwmeester D. (2008). Creating and verifying a quantum superposition in a micro-optomechanical system. New J. Phys..

[16] Abdi M., Degenfeld-Schonburg P., Sameti M., Navarrete-Benlloch C., Hartmann M.J. (2016). Dissipative optomechanical preparation of macroscopic quantum superposition states. Phys. Rev. Lett..

[17] Verhagen E., Deléglise S., Weiss S., Schliesser A., Kippenberg T.S. (2012). Quantum-coherent coupling of a mechanical oscillator to an optical cavity mode. Nature.

[18] Vitali D., Gigan S., Ferreira A., Böhm H.R., Tombesi P., Guerreiro A., Vedral V., Zeilinger A., Aspelmeyer M. (2007). Optomechanical entanglement between a movable mirror and a cavity field. Phys. Rev. Lett..

[19] Asadian A., Brukner C., Rabl P. (2014). Probing macroscopic realism via Ramsey correlation measurements. Phys. Rev. Lett..

[20] Dehghani A., Mojaveri B., Vaez M. (2020). Spin-bath dynamics in a quantum resonator-qubit system: Effect of a mechanical resonator coupled to a central qubit. Int. J. Theor. Phys..

[21] Kolkowitz S., Jayich A.C.B., Unterreithmeier Q.P., Bennett S.D., Rabl P., Harris J.G.E., Lukin M.D. (2012). Coherent sensing of a mechanical resonator with a single-spin qubit. Science.

[22] Lin Q., Rosenberg J., Jiang X., Vahala K.J., Painter O. (2009). Mechanical oscillation and cooling actuated by the optical gradient force. Phys. Rev. Lett..

[23] Mercadé L., Martín L.L., Griol A., Navarro-Urrios D., Martínez A. (2020). Microwave oscillator and frequency comb in a silicon optomechanical cavity with a full phononic bandgap. Nanophotonics.

[24] Krause A.G., Winger M., Blasius T.D., Lin Q., Painter O. (2012). A high-resolution microchip optomechanical accelerometer. Nat. Photonics.

[25] Forstner S., Prams S., Knittel J., van Ooijen E.D., Swaim J.D., Harris G.I., Szorkovszky A., Bowen W.P., Rubinsztein-Dunlop H. (2012). Cavity optomechanical magnetometer. Phys. Rev. Lett..

[26] Jayich A.M., Sankey J.C., Zwickl B.M., Yang C., Thompson J.D., Girvin S.M., Clerk A.A., Marquardt F., Harris J.G.E. (2008). Dispersive optomechanics: A membrane inside a cavity. New J. Phys..

[27] Mumford J., O’Dell D.H.J., Larson J. (2015). Dicke-type phase transition in a multimode optomechanical system. Ann. Phys..

[28] Baumann K., Mottl R., Brennecke F., Esslinger T. (2011). Exploring symmetry breaking at the Dicke quantum phase transition. Phys. Rev. Lett..

[29] Wurl C., Alvermann A., Fehske H. (2016). Symmetry-breaking oscillations in membrane optomechanics. Phys. Rev. A.

[30] Miri M.-A., Verhagen E., Alù A. (2017). Optomechanically induced spontaneous symmetry breaking. Phys. Rev. A.

[31] Birman J.L., Nazmitdinov R.G., Yukalov V.I. (2013). Effects of symmetry breaking in finite quantum systems. Phys. Rep..

[32] Reslen J., Quiroga L., Johnson N.F. (2005). Direct equivalence between quantum phase transition phenomena in radiation-matter and magnetic systems: Scaling of entanglement. Europhys. Lett..

[33] Vidal J., Dusuel S. (2006). Finite-size scaling exponents in the Dicke model. Europhys. Lett..

[34] Plastina F., Liberti G., Carollo A. (2006). Scaling of Berry’s phase close to the Dicke quantum phase transition. Europhys. Lett..

[35] Liberti G., Piperno F., Plastina F. (2010). Finite-size behavior of quantum collective spin systems. Phys. Rev. A.

[36] Kónya G., Nagy D., Szirmai G., Domokos P. (2012). Finite-size scaling in the quantum phase transition of the open-system Dicke model. Phys. Rev. A.

[37] Bhaseen M.J., Mayoh J., Simons B.D., Keeling J. (2012). Dynamics of nonequilibrium Dicke models. Phys. Rev. A.

[38] Gelhausen J., Buchhold M. (2018). Dissipative Dicke model with collective atomic decay: Bistability, noise-driven activation, and the nonthermal first-order superradiance transition. Phys. Rev. A.

[39] Reiter F., Nguyen T.L., Home J.P., Yelin S.F. (2020). Cooperative breakdown of the oscillator blockade in the Dicke model. Phys. Rev. Lett..

[40] Fuchs S., Ankerhold J., Blencowe M., Kubala B. (2016). Non-equilibrium dynamics of the Dicke model for mesoscopic aggregates: Signatures of superradiance. J. Phys. B At. Mol. Opt. Phys..

[41] Zhiqiang Z., Lee C.H., Kumar R., Arnold K.J., Masson S.J., Parkins A.S., Barret M.D. (2017). Nonequilibrium phase transition in a spin-1 Dicke model. Optica.

[42] Klinder J., Ke H., Wolke M., Mathey L., Hemmerich A. (2015). Dynamical phase transition in the open Dicke model. Proc. Natl. Acad. Sci. USA.

[43] Gruner T., Welsch D.-G. (1996). Quantum-optical input-output relations for dispersive and lossy multilayer dielectric plates. Phys. Rev. A.

[44] Dicke R.H. (1954). Coherence in spontaneous radiation processes. Phys. Rev..

[45] Hepp K., Lieb E.H. (1973). On the superradiant phase transition for molecules in a quantized radiation field: The Dicke maser model. Ann. Phys..

[46] Wang Y.K., Hioe F.T. (1973). Phase transition in the Dicke model of superradiance. Phys. Rev. A.

[47] Kirton P., Roses M.M., Keeling J., Della Torre E.G. (2018). Introduction to the Dicke Model: From Equilibrium to Nonequilibrium, and Vice Versa. Adv. Quantum Technol..

[48] Shen L., Shi Z., Yang Z., Wu H., Zhong Z., Zheng S. (2020). A similarity of quantum phase transition and quench dynamics in the Dicke model beyond the thermodynamic limit. EPJ Quantum Technol..

[49] Chen Q.-H., Zhang Y.-Y., Liu T., Wang K.-L. (2008). Numerically exact solution to the finite-size Dicke model. Phys. Rev. A.

[50] Yang M.-F. (2007). Ground-state fidelity in one-dimensional gapless models. Phys. Rev. B.

[51] Gu S.-J., Kwok H.-M., Ning W.-Q., Lin H.-Q. (2008). Fidelity susceptibility, scaling, and universality in quantum critical phenomena. Phys. Rev. B.

[52] Liu T., Zhang Y.-Y., Chen Q.-H., Wang K.-L. (2009). Large-scaling behavior of the ground-state energy, fidelity, and the order parameter in the Dicke model. Phys. Rev. A.

[53] Dey A., Mahapatra S., Roy P., Sarkar T. (2012). Information geometry and quantum phase transitions in the Dicke model. Phys. Rev. E.

[54] Bastarrachea-Magnani M.A., Castaños O., Nahmad-Achar E., López-Peña R., Hirsch J.G.J. (2014). Fidelity, susceptibility and critical exponents in the Dicke model. Phys. Conf. Ser..

[55] Nagy Á., Romera E. (2015). Relative Rényi entropy and fidelity susceptibility. Europhys. Lett..

[56] Wei B.-B., Lv X.-C. (2018). Fidelity susceptibility in the quantum Rabi model. Phys. Rev. A.

[57] Sachdev S. (2011). Quantum Phase Transitions.

[58] Robertson H.P. (1929). The uncertainty principle. Phys. Rev..

[59] Schrdinger E. (1930). About Heisenberg Uncertainty Relation. arXiv.

[60] Song L., Yan D., Ma J., Wang X. (2009). Spin squeezing as an indicator of quantum chaos in the Dicke model. Phys. Rev. E.

[61] Bakemeier L., Alvermann A., Fehske H. (2012). Quantum phase transition in the Dicke model with critical and noncritical entanglement. Phys. Rev. A.

[62] Xu H., Kemiktarak U., Fan J., Ragole S., Lawall J., Taylor J.M. (2017). Observation of optomechanical buckling transitions. Nat. Commun..

[63] Heiss W.D., Scholtz F.G., Geyer H.B.J. (2005). The large N behaviour of the Lipkin model and exceptional points. Phys. A Math. Gen..

[64] Moreira T., Pellegrino G.Q., de Faria J.G.P., Nemes M.C., Camargo F., de Toledo Piza A.F.R. (2008). Entanglement and classical instabilities: Fingerprints of electron-hole-to-exciton phase transition in a simple model. Phys. Rev. E.

[65] Perelomov A.M. (1986). Generalized Coherent States and Their Applications.

[66] de Oliveira F.A.M., Kim M.S., Knight P.L., Buzek V. (1990). Properties of displaced number states. Phys. Rev. A.

[67] Bastarrachea-Magnani M.A., Hirsch J. (2011). Numerical solutions of the Dicke Hamiltonian. Rev. Mex. Fís..

